# The phylogeny, phylogeography, and diversification history of the westernmost Asian cobra (Serpentes: Elapidae: *Naja oxiana*) in the Trans‐Caspian region

**DOI:** 10.1002/ece3.7144

**Published:** 2020-12-22

**Authors:** Elmira Kazemi, Masoud Nazarizadeh, Faezeh Fatemizadeh, Ali Khani, Mohammad Kaboli

**Affiliations:** ^1^ Department of Environment Faculty of Natural Resources and Environment Science and Research Branch Islamic Azad University Tehran Iran; ^2^ Department of Parasitology Faculty of Science University of South Bohemia České Budějovice Czech Republic; ^3^ Institute of Parasitology Biology Centre CAS, v.v.i. České Budějovice Czech Republic; ^4^ Department of Environmental Science Faculty of Natural Resources University of Tehran Karaj Iran; ^5^ Department of Environment Khorasan Razavi Mashhad Iran

**Keywords:** demographic history, *Naja oxiana*, phylogeny, phylogeography

## Abstract

We conducted a comprehensive analysis of the phylogenetic, phylogeographic, and demographic relationships of Caspian cobra (*Naja oxiana;* Eichwald, 1831) populations based on a concatenated dataset of two mtDNA genes (cyt *b* and ND4) across the species' range in Iran, Afghanistan, and Turkmenistan, along with other members of Asian cobras (*i.e.,* subgenus *Naja* Laurenti, 1768). Our results robustly supported that the Asiatic *Naja* are monophyletic, as previously suggested by other studies. Furthermore, *N*. *kaouthia* and *N. sagittifera* were recovered as sister taxa to each other, and in turn sister clades to *N. oxiana*. Our results also highlighted the existence of a single major evolutionary lineage for populations of *N. oxiana* in the Trans‐Caspian region, suggesting a rapid expansion of this cobra from eastern to western Asia, coupled with a rapid range expansion from east of Iran toward the northeast. However, across the Iranian range of *N. oxiana*, subdivision of populations was not supported, and thus, a single evolutionary significant unit is proposed for inclusion in future conservation plans in this region.

## Graphical abstract

We conducted a comprehensive analysis of the phylogenetic, phylogeographic, and demographic relationships of Caspian cobra (*Naja oxiana*) populations based on a concatenated dataset of two mtDNA genes. Our results provided strong support of phylogeny of Caspian cobras. Furthermore, our results also highlight the existence of only one major evolutionary lineage in the Trans‐Caspian region, suggesting a rapid expansion of the Caspian cobra from eastern to western Asia. We proposed one evolutionary significant unit across the Iranian range of *N. oxiana* for conservation efforts.

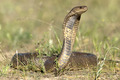

## 1 INTRODUCTION

**Figure 1:**
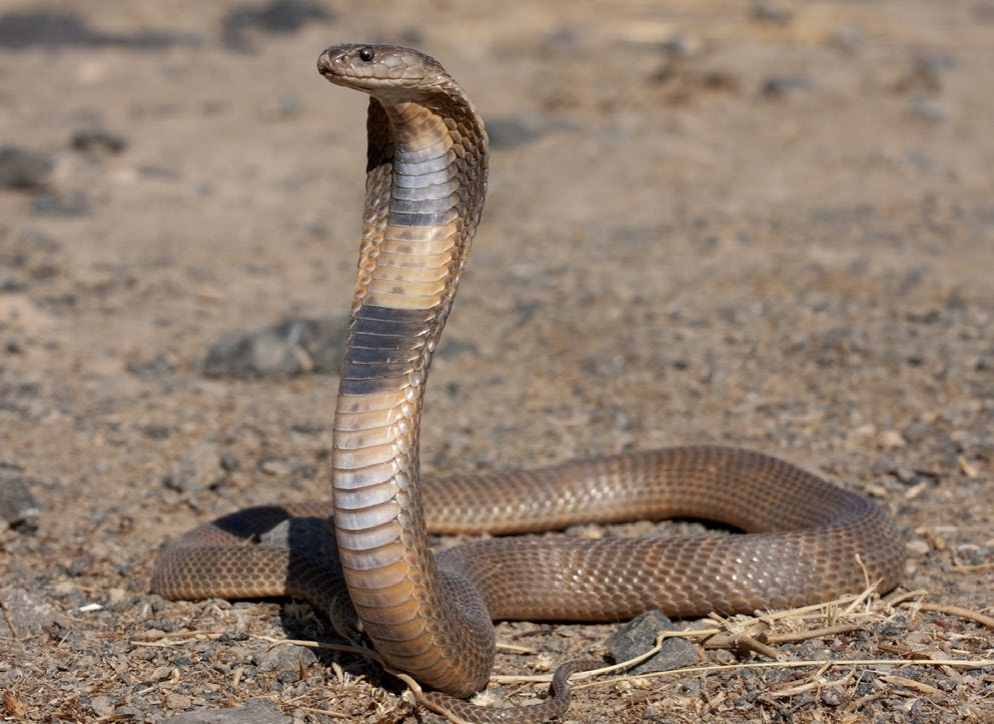
The Caspian cobra (*Naja oxiana*), aka the Central Asian cobra. This member of the family Elapidae is a nonspitter. Credit: Ali Khani

**Figure 2:**
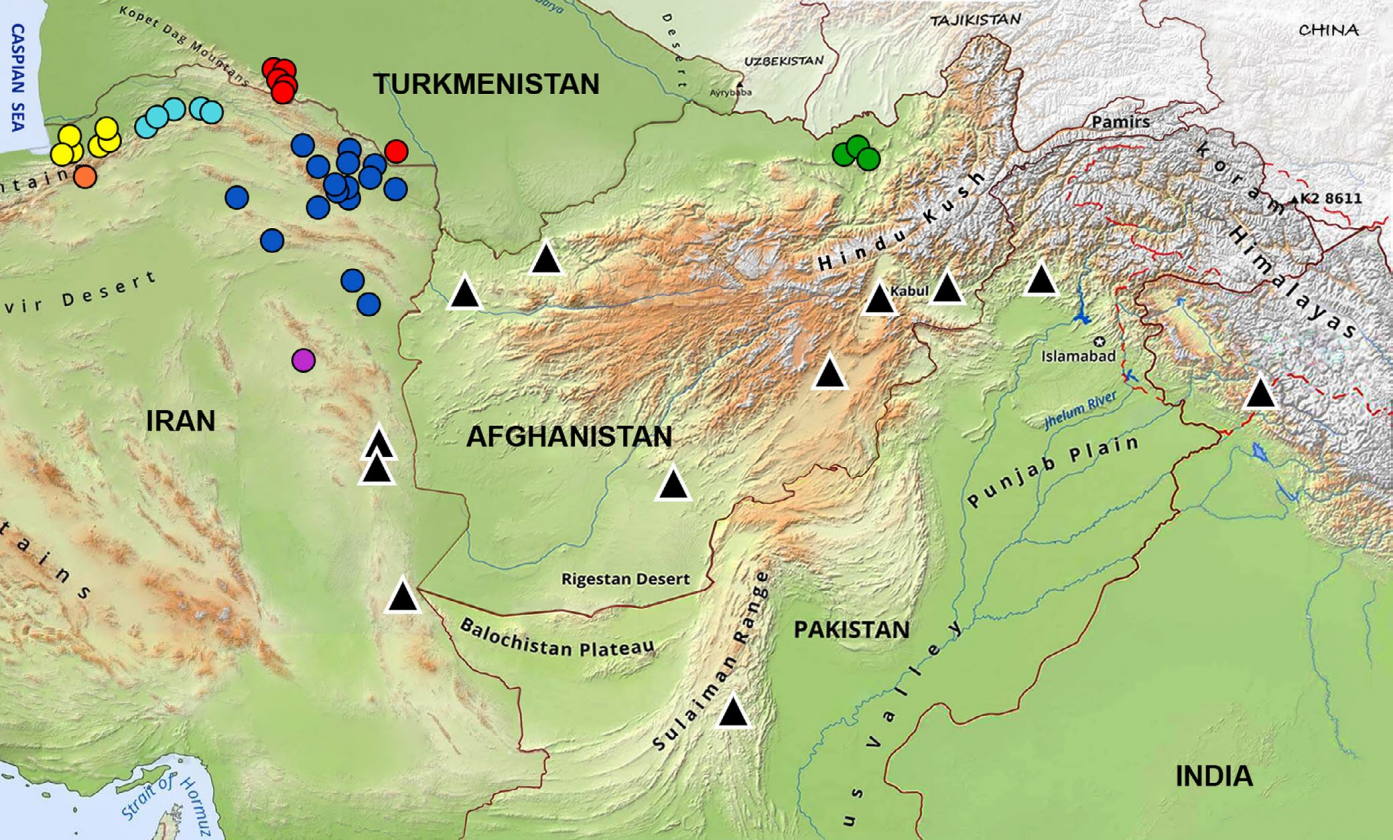
Top: map showing the distribution of the 11 cobra species of Asiatic *Naja*, ranging from Trans‐Caspia to southern and southeastern Asia. Geographic ranges were obtained from the IUCN Red List (IUCN, 2020) and Wüster (1996) and updated for some species based on new occurrence records. Blue circles represent *N. oxiana* occurrence points that lie outside the species' known range. White dotted line represents *N. sumatrana's* range boundary. Bottom: map of sampling localities of the 38 specimens from Iran, Turkmenistan, and Afghanistan. Colors correspond to sampling localities of *N. oxiana*, namely Golestan (purple), Semnan (orange), Northern Khorasan (light blue), Central Khorasan (dark blue), and Southern Khorasan (yellow) provinces of Iran, as well as Turkmenistan (red) and Afghanistan (green). Black triangles once again represent *N. oxiana's* occurrence points that lie outside the species' known range

African and Asian cobras (genus *Naja* Laurenti, 1768) comprise four subgenera (Wallach et al., 2009), including *Naja* (Laurenti, 1768), *Uraeus* (Wagler, 1830), *Boulengerina* (Dollo, 1886), and *Afronaja* subgen.nov, within which the typical form *Naja* (*Naja*) is restricted to 11 species in Asia, occurring from Trans‐Caspia to southern and southeastern Asia, throughout the East Indies, the Philippines, and the Lesser Sunda Islands (Figure 2; Slowinski & Wüster, 2000; Wüster & Broadley, 2003). This subgenus is thought to have an African origin which likely originated from a single invasion of Asia from Africa (Slowinski & Wüster, 2000; Wallach et al., 2009; Wüster et al., 2007). Fang structure is variable within the subgenus *Naja* in that, except for *N. naja* and *N. oxiana*, all other members of the subgenus have fully or partially evolved the spitting behavior (Gold et al., 2002; Lin et al., 2014; O'Shea, 2008; Wüster & Broadley, 2003; Wüster & Thorpe, 1992) (Figure 1).

The Caspian cobra *N. oxiana* (Eichwald, 1831) has the westernmost Asian distribution in this subgenus. The species occurs in relatively dry, stony habitats of the Trans‐Caspian region including areas with dispersed vegetation in northeastern Iran, Turkmenistan, Uzbekistan, Kyrgyzstan, southwestern Tajikistan, eastern and northern Afghanistan, Pakistan, and northern India (Klemmer, 1968; Rajabizadeh, 2018; Valenta, 2009). Within its distribution range, *N. oxiana* is subdivided into eastern and western populations, split by the Hindu Kush Mountains and desert zones of southern Afghanistan, southeastern Iran, and southwestern Pakistan (Wüster, 1990). However, a new population in its eastern distribution has been recently recorded in Himachal Pradesh, India, at an altitude of approximately 2,100 m (Santra et al., 2019). Based on morphological characters, Wüster (1990) suggested that the western population is relatively homogeneous but morphologically different from the eastern population. Additionally, western *N. oxiana* populations appear to lack cuneate scales, whereas eastern populations, similar to most cobras of Asiatic *Naja,* have a cuneate scale on each side.

The distribution, demography, biology, ecology, and conservation status of the Caspian cobra remain largely undescribed. Even though some molecular studies have investigated Asian and African cobras (Kazandjian et al., 2020; Lin et al., 2008, 2012, 2014; Ratnarathorn et al., 2019; Santra et al., 2019; Wallach et al., 2009; Wüster et al., 2007), the phylogenetic and phylogeographic status, as well as the evolutionary history and population structure of the Caspian cobra in the Trans‐Caspian region, are still poorly known. The only study conducted on the genetic structure and phylogeny of the Caspian cobra in Iran, using 589 base pairs (bp) of the mitochondrial D‐loop region, revealed low genetic diversity and unstructured populations of the Iranian Caspian cobra (Shoorabi et al., 2017). Yet, it is unclear when or how the species dispersed into northeastern Iran, expanding its range from the dry and semidry mountainous habitats bordering Iran, Afghanistan, and Turkmenistan to the hot and humid plains of Golestan Province, southeast of the Caspian Sea. This species is known as rare and vulnerable in Iran (Darvish & Rastegar‐Pouyani, 2012) and is listed in Appendix II of CITES; however, it has been designated as “data deficient” (DD) under the criteria of the International Union for Conservation of Nature (IUCN). For nearly a century, this cobra has been intensively harvested for venom extraction by the largest Iranian venom manufacturing center, the Razi Institute for Serum and Vaccine Research (Darvish & Rastegar‐Pouyani, 2012), which is perceived to be the main cause for the dramatic decline in its wild populations.

Here, we aimed to ascertain the first insight into the phylogeny and phylogeography of the Caspian cobra across its distribution range in the Trans‐Caspian region, using the Cytochrome *b* (cyt *b*) and NADH dehydrogenase subunit 4 (ND4) markers. We sought to (a) demonstrate the phylogenetic relationships of the Caspian cobra and other members of Asian cobras (subgenus *Naja*), (b) uncover the phylogenetic processes, including the timing and mechanisms of the species' colonization from southwest Asia into Iran and subsequently to the plains of the Caspian Sea, and (c) delineate the evolutionary lineages of the Caspian cobra across its geographical range in the Trans‐Caspian region and define the species' ESUs in Iran.

## 2 MATERIAL AND METHODS

### 2.1 Sample collection

Sampling permits were issued by the Department of Environment of Iran (license numbers: 94/43027 and 94/17701). Tissue sampling of 38 *N. oxiana* specimens was done from western populations of the species in Turkmenistan, Afghanistan, and Iran during 2014–2016. Samples of each specimen consisted of three scale clippings taken from the outer layer of the ventral scales, except for 10 muscle tissue samples acquired from museums of Turkmenistan and Afghanistan (Figure 2). After sampling, the cobras were immediately released back at their capture location. All procedures were carried out following the relevant guidelines and approved regulations. Also, we obtained 47 sequences of 11 Asian cobras (two samples of *N. oxiana*, seven samples of *N. atra* CANTOR, 1842; three samples of *N. naja* LINNAEUS, 1758; six samples of *N. siamensis* LAURENTI, 1768; four samples of *N. sumatrana* MÜLLER, 1887; five samples of *N. philippinensis* Taylor, 1922; three samples of *N. samarensis* Peters, 1861; two samples of *N. sagittifera* WALL, 1913; three samples of *N. sputatrix* F. Boie, 1827; nine samples of *N. kaouthia* LESSON, 1831; and three samples of *N. mandalayensis* Slowinski & Wüster, 2000) from GenBank (Appendix A).

### 2.2 DNA isolation, amplification, and fragment analysis

For genomic DNA extraction, we used the standard phenol/chloroform protocol. Two fragments of the mtDNA containing 1,060 bp of the cyt *b* and 699 bp of the ND4 loci were amplified using four pairs of modified primers (ND4/Leu (Arevalo et al., 1994) and L14910/H16064 (Burbrink et al., 2000; Table 1). For PCR mix preparation, a final volume of 25 µl consisting of 1 µl of primer, 12.5 µl of Taq Mix (2×), 5 ng of template DNA, and 5.5 µl of double‐distilled H_2_O was used. Typical amplification conditions involved a 5‐min initial denaturation step at 95°C and 35 cycles at 95°C for 30 s. Next, primer annealing was done at 58°C (cyt *b*) and 52°C (ND4) for 30 s, and 72°C for 1 min, followed by a 5‐min primer extension step at 72°C. PCR products were then analyzed and verified by agarose electrophoresis. Sequencing of the purified products was performed in an ABI PRISM 3730xl automatic sequencer (Korea Genomics, Bioneer).

**Table 1:** Modified sequences of the primers used for PCR and/or sequencing

Primers	Sequences	Sources
cyt *b*‐F1 cyt *b*‐R1	GTCCTGCGGCCTGAAAAACCACCGTTGT CTTTGGTTTACAAGAACAATGCTTTG	Modified from Burbrink et al. (2000)
cyt *b*‐F2 cyt *b*‐R2	AACAGCCTTCTTCGGATACG AATCGGGTGAGGGTTGGG	
ND4‐F1 ND4‐R1	CACCTTTGACTACCCAAAGCCCACGTCGAAGC CCTTACTTTTACTGGGATTTGCACCA	Modified from Arevalo et al. (1994)
ND4‐F2 ND4‐R2	CCTCATCAGCACTATTCTGCCTAGC TATAAGTAGGTGTTCTCGTGAGTG	

### 2.3 Sequence analysis

The SeqScape version 2.6 software (Applied Biosystems) was used for editing the sequences, and ClustalW via MEGA version 5 (Tamura et al., 2011) was applied for generating a multiple sequence alignment. Sequences of protein coding genes were translated to search for possible stop codons due to pseudogene production. The substitution saturation test (Xia et al., 2003) was conducted using DAMBE version 6.0.4 (Xia, 2013). Genetic diversity (nucleotide and haplotype diversities), polymorphic sites, and haplotype numbers were measured using DnaSP version 5.0 (Librado & Rozas, 2009). Genetic distances and composition of nucleotides were examined based on the uncorrected pairwise genetic distances with 1,000 bootstraps using MEGA version 5 (Tamura et al., 2011). Furthermore, we computed all pairwise distances, between and among groups, nucleotide composition and translation/transversion ratios using MEGA version 5 (Tamura et al., 2011). We also performed a partition homogeneity test to evaluate differences in phylogenetic information content among the two mitochondrial fragments via the Shimodaira–Hasegawa (SH; Shimodaira & Hasegawa, 1999) and the approximate unbiased (AU) tests in PAUP* version 4.0b10 (Swofford, 2002), conducted with heuristic searches of 1,000 replicates.

### 2.4 Phylogenetic analyses

We constructed a concatenated dataset of two fragments of mtDNA including 1,759 bp in length for 38 samples of *N. oxiana* from the western population in Iran, Turkmenistan, and Afghanistan, two samples of *N. oxiana* from the eastern population downloaded from GenBank (1,309 and 1,312 bp in length), 45 samples of the other ten Asiatic *Naja* species from GenBank, and finally two samples of African cobras (*N. nivea* and *N. haje*) downloaded from GenBank, which were used as out‐groups. Phylogenetic tree reconstructions were done based on the Bayesian inference (BI) and maximum‐likelihood (ML) approaches. To determine the optimal substitution models and partitioning schemes at first, second, and third codon positions, PartitionFinder version 1.1.1 (Lanfear et al., 2012) was adopted for both the BI and ML analyses. Partitioning of three different data subsets was detected: (a) cyt *b*‐pos1/ND4‐pos1, (b) cyt *b*‐pos2/ND4‐pos2, and (c) cyt *b*‐pos3/ND4‐pos3, for which the best fitting models were found to be TRN+I+Γ, GTR+I+Γ, and HKY+I, respectively.

We used IQ‐Tree version 1.6.8 (Nguyen et al., 2014) to build an ML tree using 1,000 nonparametric bootstrap replicates and estimate support of the tree topology (Hoang et al., 2018). Furthermore, a Bayesian phylogenetic reconstruction was performed using the selected scheme in MrBayes version 3.2.4 (Ronquist & Huelsenbeck, 2003). For Bayesian inference, four Markov chains Monte Carlo runs (one cold and three heated chains (MC^3^)) were simultaneously used for 40 million generations with two replicates, sampling the trees every 1000th generation and removing the first 25% burn‐in trees. By combining the remaining trees, we obtained a 50% majority rule consensus tree. Tracer version 1.7 was implemented to visualize the results and to measure stationarity and convergence of the chains (Rambaut et al., 2018). Bayesian posterior probabilities were computed to check for support of the Bayesian tree branches.

### 2.5 Analyses of population genetic structure

To detect genetic clusters in the concatenated mtDNA dataset, we applied the Bayesian inference of genetic structures of populations in BAPS version 6.0 (Corander et al., 2013). A range of 1–20 was considered for values of the number of clusters (K). Moreover, two haplotype networks were constructed to reveal haplotype relationships among the 11 Asian cobras. To reconstruct the phylogenetic relationships among Asian cobras, SplitsTree version 4.6 (Huson & Bryant, 2006) was used and a neighbor‐net network was generated using the uncorrected patristic distances with 1,000 bootstrap replicates, and to plot haplotype relationships within *N. oxiana* populations, TCS algorithm was used in PopArt (Leigh & Bryant, 2015).

### 2.6 Molecular dating estimates

To determine divergence times, BEAST version 1.8.0 (Drummond & Rambaut, 2007) was implemented using three fossil calibration points recommended by Head et al. (2016) including (a) Viperinae: the divergence between Crotalinae and Viperinae dating back to at least 20 Mya (Szyndlar & Rage, 1990; Wüster et al., 2008). For this node, we used a lognormal prior with 20 Mya as zero offset, a standard deviation of 1 and a mean of 1, providing a 95% confidence interval of 20.52–34.08; and (b) *Bungarus*: following the divergence time between *Bungaurs bungaroides*, *B. flaviceps*, and the *B. fasciatus* clade around 10.215 Mya (Barry et al., 2002), providing a confidence interval of 10.74–24.3. To constrain this node, we used a standard deviation of 1, a mean of 1 and an offset zero of 17.0 Mya. (c) *Naja*: We used *N. romani* fossil (Hoffstetter, 1939) which marks the divergence between *Naja* and *Haemachatus* dating back at 17 Mya modeled using lognormal prior with a 95% confidence interval of 17.52–31.08.

In search of the fittest partitioning schemes for our data and models of evolution for molecular dating analyses, PartitionFinder version 1.1.1 (Lanfear et al., 2012) was utilized. Also, a birth–death process was applied as it is more fitting for a multispecies sequence dataset (Drummond & Rambaut, 2007). Fitness of the molecular clocks (strict, exponential relaxed, and lognormal relaxed) was assessed based on the Bayes factor support value (Brandley et al., 2005) estimated by Tracer version 1.5. For the dating analyses, we ran two independent Markov chains for 40 million generations, sampling every 1000th generation and removing the first 25% burn‐in trees. To check for convergence of MCMC runs, stationary distribution of the chains, adequate chain mixing, and effective sample sizes of parameters, we used Tracer version 1.5.

We added 29 sequences from GenBank to our concatenated dataset including: two species of African cobras (*N. nivea* and *N. haje*), one species of *Hemachatus,* three species of *Bungarus,* three species of *Macrovipera,* eight species of *Montivipera,* two species of *Vipera,* four species of *Porthidium,* four species of *Crotalus,* and two species of *Sistrurus* for our molecular dating (see Appendix A).

### 2.7 Neutrality tests and demographic analyses

The Extended Bayesian Skyline Plot (EBSP; Ho & Shapiro, 2011) was created in Beast version 2.4.7, allowing us to estimate the demographic history of *N. oxaina* using the concatenated mtDNA dataset. Strict molecular clocks were used with a mutation rate of 0.0065/site/myr (Lin et al., 2014). The MCMC chain was set to a total of 500 million steps, and the Markov chain was sampled every 10,000 steps. To check for convergence of MCMC runs and examine effective sample sizes (>200), Tracer version 1.5 was utilized. Bayesian skyline plots were created using the EBSP R function (Heled & Drummond, 2008).

The demographic signatures of population expansion in *N. oxiana* were inferred using mismatch distributions. We calculated sums of squared deviations (SSD) and Harpending's raggedness index (RI) in Arlequin version 3.1 (Excoffier & Lischer, 2010) for comparison of observed distributions with expected distributions under the expansion model. Additionally, tests of Fu's *F*s statistics (Fu, 1997) and Tajima's *D* (Tajima, 1989) were estimated to test equilibrium of the population in Arlequin version 3.1 (Excoffier & Lischer, 2010). Negative statistics signify an excess of low frequency alleles, which suggests size expansion and/or purifying selection of a population (Tajima, 1989).

## 3 RESULTS

### 3.1 Phylogenetic reconstructions

Based on our phylogenetic reconstruction analyses, our concatenated dataset of 38 sequences of the western population of *N. oxiana* revealed 1,721 invariable sites, 34 singletons, and 4 parsimony informative sites, out of 1,759 bp aligned positions. No indels (insertions/deletions) or stop codons were detected in the alignment. The sequences had the following base compositions: T = 26.0%, C = 31.7%, A = 31.1%, and G = 11.2%. Additionally, nucleotide (pi) and haplotype (h) diversities were estimated to be 0.0075 and 0.75 for *N. oxiana*, respectively.

According to the HKA tests, our mtDNA polymorphisms showed no departure from expectations of the neutral model of evolution. Results of the HKA tests revealed nonsignificant differences from neutrality for cyt *b* (44 sequences, χ^2^ = 0.116, *p* = .73) and ND4 (38 sequences, χ^2^ = 0.015, *p* = .83). Furthermore, saturation analyses showed that the two mtDNA genes were suitable for phylogenetic analyses, with index values of the observed substitution saturation (*ISS*) being significantly smaller than the critical (*ISSc*) values (not shown here), signifying minimal substitution saturation. Also, the AU and SH partition homogeneity tests were nonsignificant (*p* > .05), verifying that the genes could be combined for phylogenetic analyses.

**Figure 3:**
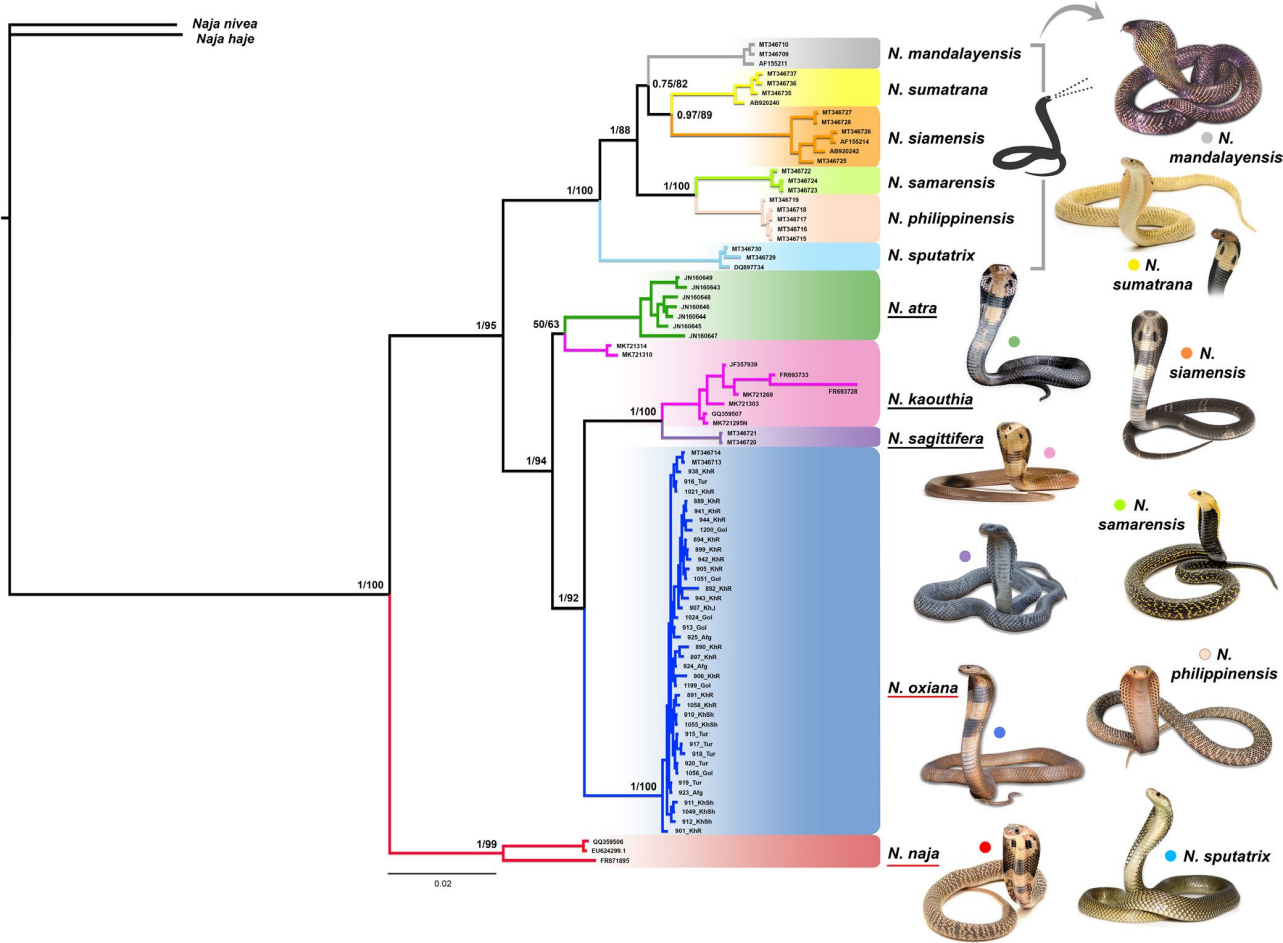
Bayesian tree reconstructed using the concatenated mtDNA dataset (branching pattern and clade positions are concordant with the ML tree), using two out‐groups (*N. nivea* and *N. haje*). Nodal support at each node indicates BI (left) and ML (right) support values. The six species in square bracket at the top are spitters, the three species underlined in black (*N. atra*, *N. kaouthia*, *N. sagittifera*) are possible spitters, and the two species underlined in red at the bottom (*N. oxiana* and *N. naja*) are nonspitters

Both ML and BI phylogenetic trees generated congruent branching patterns. In both reconstructions, *N*. *naja* was strongly supported as the basal lineage for the remaining lineages by high BI posterior probability (1.00) support and ML bootstrap value (99). Furthermore, southeastern Asian (*N. siamensis, N. sumatrana, N. philippinensis, N. samarensis, N. sputatrix,* and *N. mandalayensis*) and western, central, southern, and eastern Asian (*N*. *oxiana, N*. *kaouthia*, *N. sagittifera,* and *N*. *atra*) cobras were separated into well‐diverged clades supported by high BI (1.00) and ML (95) values. However, we neither found support for significant divergence between western and eastern populations of *N. oxiana*, as sequences from the eastern population (MT346713 and MT346714) were nested within the western population, nor any genetic differentiation among *N. oxiana* populations in the Trans‐Caspian region (northeastern Iran, Turkmenistan, and Afghanistan), as these populations formed a single well‐supported clade with the other clades of Asian cobras (1.00 BI and 100 Ml). Moreover, our finding of *N*. *kaouthia* and *N. sagittifera* being recovered as sister taxa (1.00 BI and 100 Ml), and in turn as sister clades to *N. oxiana* with high support values (1.00 BI and 92 Ml) (Figure 3) is new.

### 3.2 Haplotype networks

**Figure 4:**
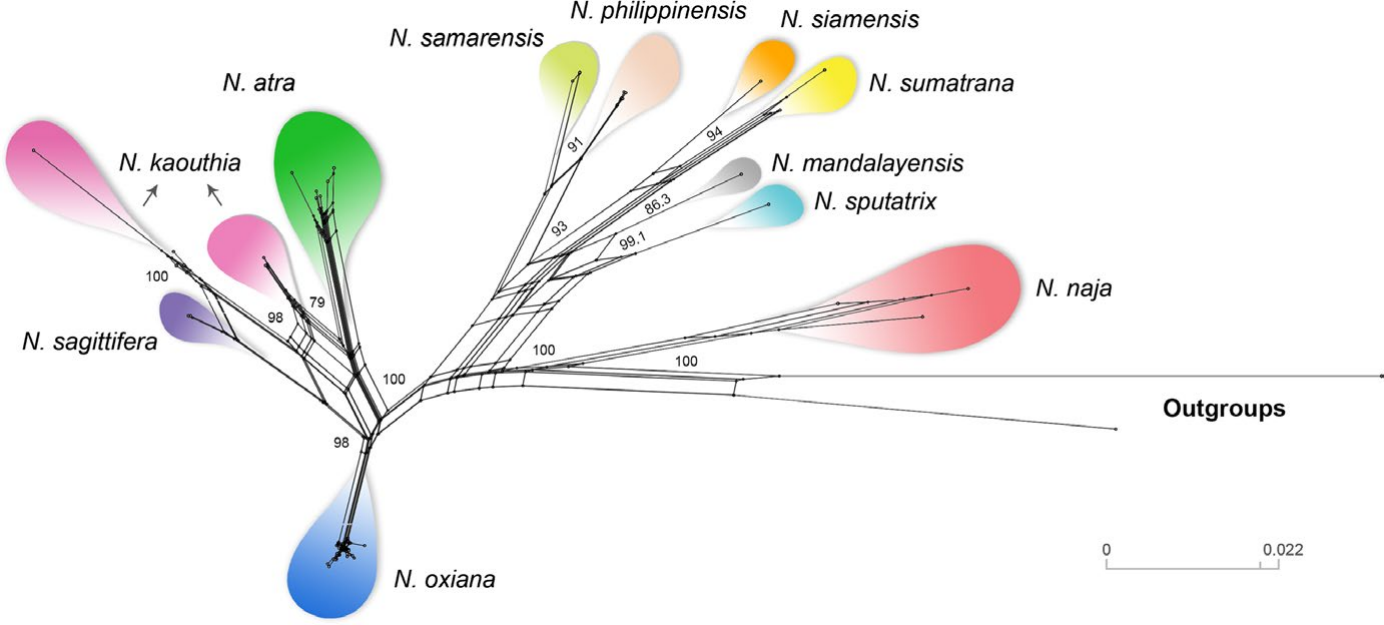
SplitsTree network using a 1,759‐bp concatenated dataset (85 sequences) detected 12 distinct haplogroups within Asian cobras, in accordance with the phylogenetic tree. *N. nivea* and *N. haje* were used as out‐groups. Colors correspond to those shown in Figure 2 (top) and Figure 3

We detected 23 haplotypes for *N. oxiana* using 38 novel sequences. The haplotype network plotted by SplitsTree v4.6 (Figure 4) using the concatenated mtDNA matrix (40 samples of *N. oxiana* and 45 samples of the other Asian cobras) detected 12 distinct haplogroups within Asian cobras, in accordance with the phylogenetic tree. Based on the phylogenetic network, *N. kaouthia* and *N. sagitifera* separated from each other with high statistical value (100 bootstrap support) and diverged from *N. oxiana* with 100 bootstrap value. Moreover, these three haplogroups differed from *N. atra* and *N. kaouthia*‐north lineage with 98% bootstrap value. All these haplogroups separated from southeastern Asian cobras including *N. samarensis*, *N. philippinensis*, *N. sumatrana*, *N. siamensis*, *N. manadalayensis*, and *N. sputatrix* with 100% bootstrap support. In addition, *N. naja* showed a divergence from all Asiatic *Naja* with high bootstrap value (100%).

**Figure 5:**
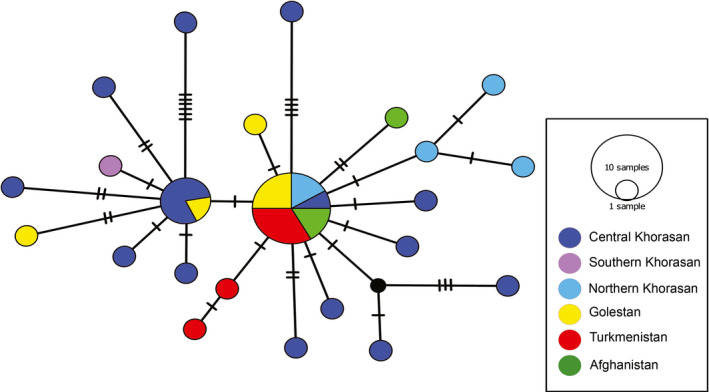
Haplotype network of *N. oxiana* from the Trans‐Caspian region (38 sequences). The central haplotype includes samples from Central Khorasan, Northern Khorasan, Golestan, Turkmenistan, and Afghanistan. Mutational steps are indicated by dash symbols along each line connecting haplotypes and branch lengths roughly correspond to mutation steps. Circle sizes correlate with haplotype frequencies. Black circles represent extinct or unsampled haplotypes. Colors correspond to those shown in Figure 2 (bottom)

**Figure 6:**
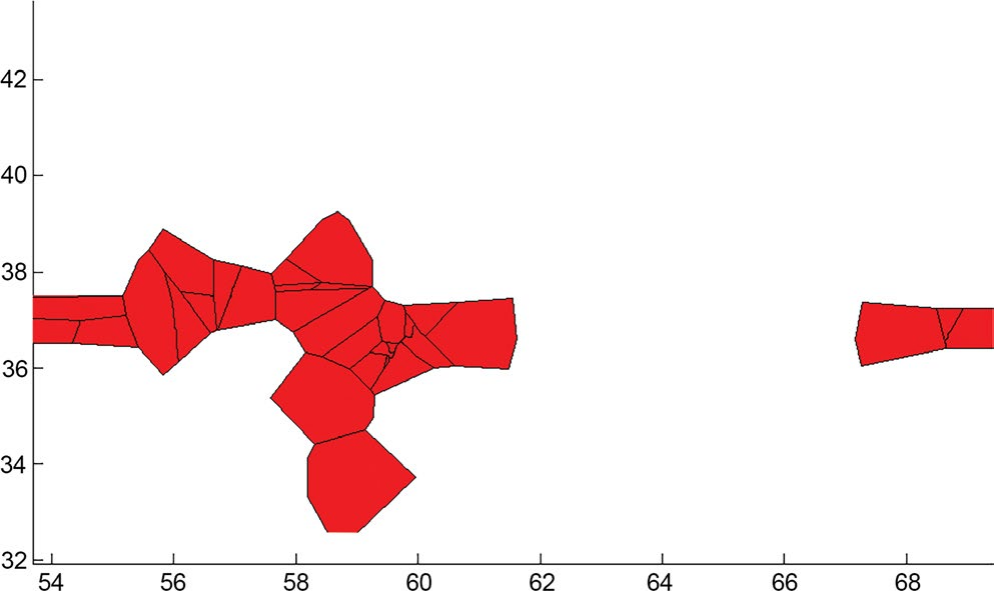
Bayesian spatial clustering for groups of individuals of *N. oxiana* performed in BAPS. The mixture analysis was set for K = 1 (one cluster)

Furthermore, using the concatenated mtDNA matrix of *N. oxiana* and in line with the phylogenetic reconstructions, TCS version 1.21 found no significant haplotype clusters from Afghanistan, Turkmenistan, and northeastern Iran (Figure 5). All populations were accompanied by multiple shared haplotypes, connected to each other by small numbers of mutational steps (1–6 steps). In addition, the BAPS analysis placed *N. oxiana* populations into one cluster (Figure 6), congruent with the haplotype network.

### 3.3 Divergence times

**Figure 7:**
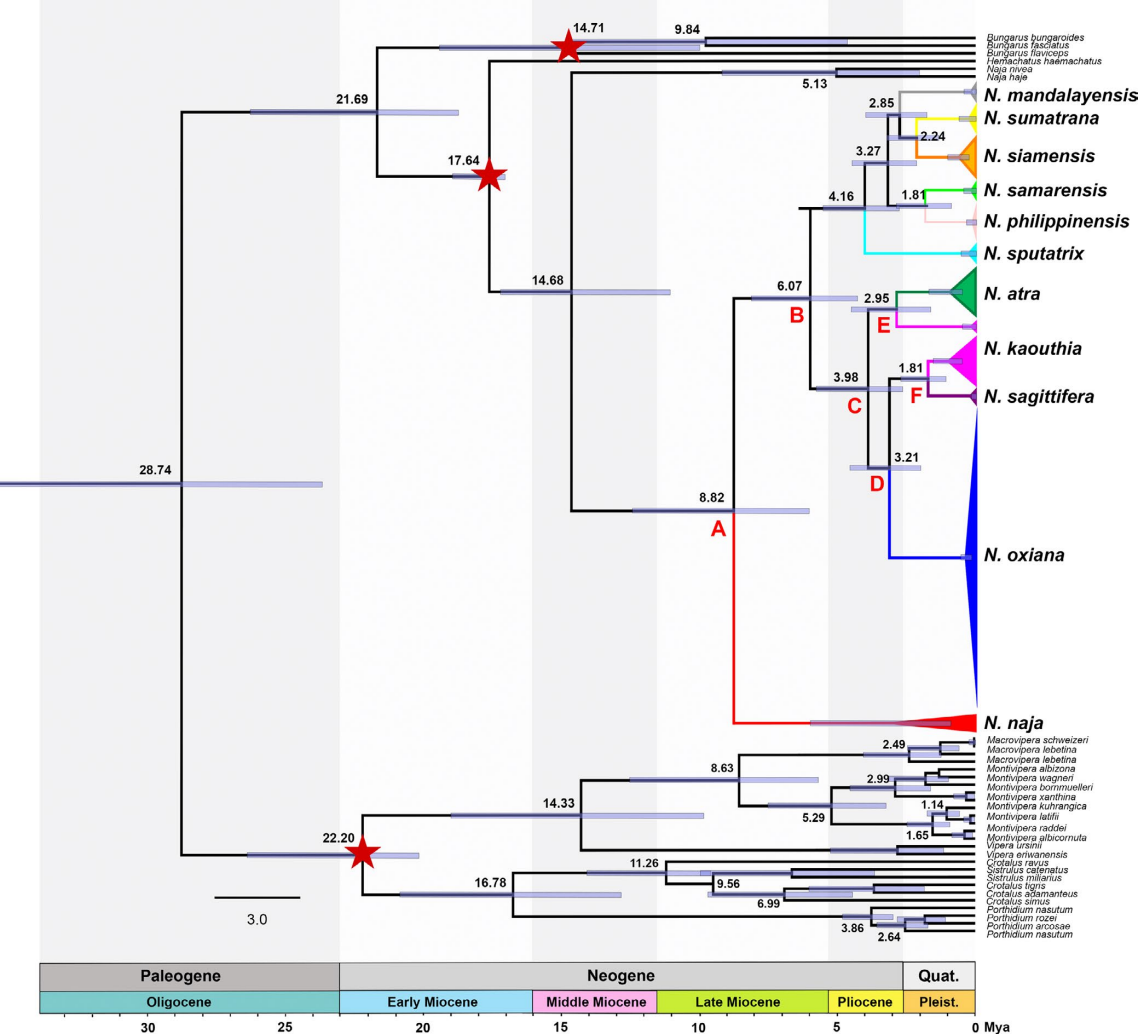
Chronogram resulting from dating analyses using the concatenated mtDNA dataset (cyt *b* + ND4) with 116 sequences (40 sequences from *Naja* oxiana, 47 sequences from the other 10 Asiatic cobras, and 29 sequences from 9 genera as out‐groups), generated by BEAST version 1.8.0 (Drummond & Rambaut, 2007). Branch numbers display times of divergence. Colors correspond to lineage colors in Figure 2 (top), Figure 3, and Figure 4. A–F refer to divergence nodes within Asiatic cobras. The three calibration points are indicated by red stars

Analyses of divergence placed the basal cladogenesis among the Asian cobras in the late Miocene (Tortonian Age) at approximately 8.82 Mya (95% HPD: 5.96–11.99 Mya; Figure 7: node A), with the nonspitting *N. naja* (Wüster & Thorpe, 1992) emerging as the basal clade of all Asian cobras. Subsequent cladogenetic events dating to late Miocene (Messinian Age) at ∼6.07 Mya (95% HPD: 4.21–8.02 Mya; Figure 7: node B) isolated the ancestor of spitting cobras of southeastern Asia (*N. siamensis, N. sumatrana, N. philippinensis, N. samarensis, N. sputatrix,* and *N. mandalayensis*) from non/occasional spitters (*N. oxiana, N. kaouthia, N. atra*, and *N. sagittifera*). Later cladogenetic events between *N. atra* and western‐central‐southern Asian cobras (*N. oxiana, N. kaouthia*, and *N. sagittifera*) dated to early Pliocene (Zanclean Age) (3.98 Mya, 95% HPD: 2.56–5.49 Mya; Figure 7: node C), whereas the divergence between *N. oxiana* and its sister taxa, *N. kaouthia* and *N. sagittifera,* took place during late Pliocene (Piacenzian Age) (3.21 Mya, 95% HPD: 1.94–4.48 Mya; Figure 7: node D). However, our molecular dating showed that a cladogenetic event dating to late Pliocene at ∼2.95 Mya (95% HPD: 1.54–4.42 Mya; Figure 7: node E) isolated the nominated “north‐eastern population of *N. kaouthia*” of Nakhon Ratchasima Province, Thailand, a paraphyletic taxon that has been recently proposed as a new *Naja* for Asia (Ratnarathorn et al., 2019). Finally, the last divergence of non/occasional spitting cobras occurred between *N. kaouthia* and *N. sagittifera* at 1.81 Mya (95% HPD: 0.99–2.61 Mya; Figure 7: node F) during early Pleistocene (Gelasian Age).

### 3.4 Demographic history

**Figure 8:**
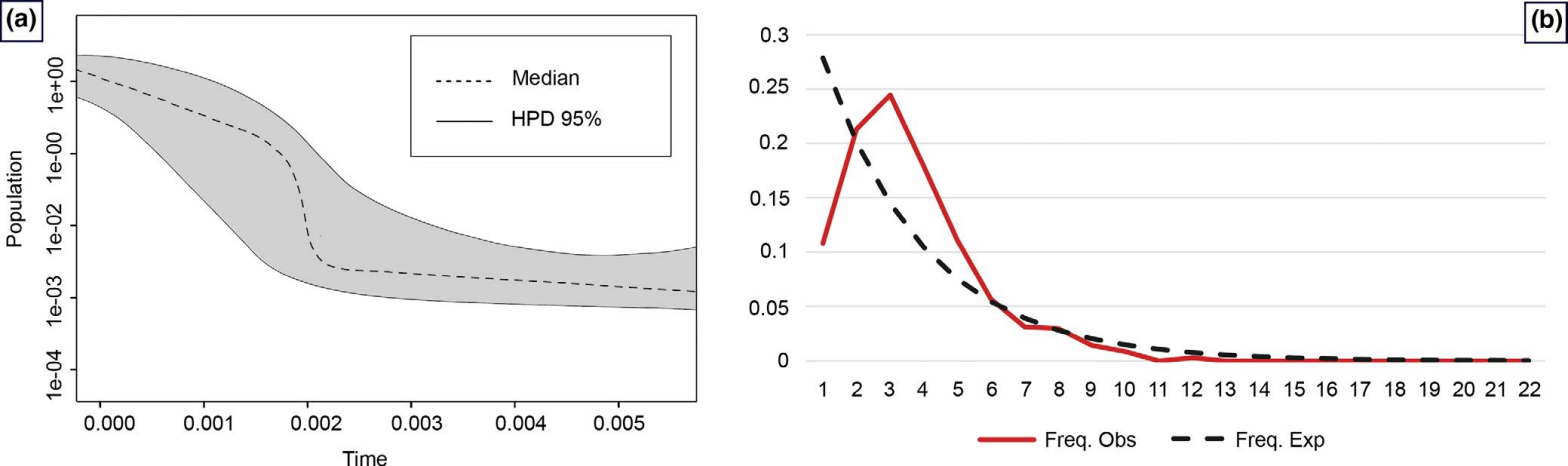
Extended Bayesian Skyline Plot, (a): *x*‐axis visualizes time before present (Mya), and *y*‐axis represents effective population sizes (Ne). Dark shaded regions depict confidence intervals (95% HPD limits), and the dashed line expresses median value for the log_10_ of Ne, (b): Mismatch distribution demonstrates the demographic history of *N. oxiana*. Dotted black line represents the expected distributions, and the solid red curve indicates distributions under a constant‐sized population assumption

EBSP indicated a population growth curve for *N. oxiana* since ∼5 Kya (Figure 8a). This plot revealed a gradual expansion since ∼5 Kya and a considerable increase in population size since 2 Kya up to the present day. Also, the demographic events estimated based on analysis of pairwise mismatch distribution of cyt *b* and ND4 haplotypes found a unimodal mismatch distribution for *N. oxiana* (Figure 8b). Furthermore, the significantly negative values of Fu' *F*s reported here (Fu' *F*s = −20.40 and Tajima *D* = −2.52) strongly suggest a recent expansion for this clade (Rogers & Harpending, 1992; Slatkin & Hudson, 1991).

## 4 DISCUSSION

### 4.1 Phylogenetic and phylogeographic patterns of the Asiatic *Naja*

The evolutionary relationships among lineages of the Asiatic *Naja* have not yet been clearly refined and addressing the issue of the evolution of spitting behavior in African and Asiatic cobras seems to be the core subject for generating a comprehensive and robust phylogenetic resolution for cobras (Wüster et al., 2007). Considering that *N. naja* and *N. oxiana* are nonspitters, *N. sputatrix*, *N. siamensis*, *N. philippinensis*, *N. samarensis*, *N. mandalayensis* and *N. sumatrana* are spitters, and *N. kaouthia*, *N. atra,* and *N. sagittifera* are non/occasional spitters (Ratnarathorn et al., 2019; Wüster & Thorpe, 1992), the concurrence of spitting and nonspitting cobras within the Asiatic *Naja* group may support the hypothesis that postulates multiple colonization events of Asia by African cobras (Ineich, 1995; Minton, 1986). However, Wüster et al. (2007) argued against this hypothesis and proposed that Asiatic *Naja* originated from a single invasion of Asia from Africa.

Barbour (1922) suggested that spitting evolved in African cobras to defend against large ungulates on their paths in African grasslands. This hypothesis, however, was criticized by Wüster et al. (2007) as spitting behavior evolved earlier than the first emergence of the great grasslands of Africa and therefore before the appearance of large ungulates in these habitats. Also, spitting cobras of the Asian *Naja* were at no risk of facing ungulate herds due to their forest‐like habitat type in southeastern Asia. Wüster et al. (2007) argue that cobras' spitting behavior may have evolved three times and their phylogeographic patterns appear to be the result of a complex interplay of geological and ecological factors. Recently, Kazandjian et al. (2020) showed that spitting behavior in cobras has evolved independently in three spitting lineages as a defensive mechanism. Their results demonstrated that venom of cobras from these lineages was endowed with an upregulation of the activity of phospholipase A2 (PLA2) enzyme, a common toxin in venom of snakes, which triggers the activity of venom cytotoxins resulting in the activation of mammalian sensory neurons, thereby causing increased pain. Based on their divergence time estimates, spitting behavior possibly evolved following the appearance of bipedal hominids in African grasslands and later in Asia. Nevertheless, at present, we have no explanation for why the nonspitting *N. naja* forms the basal lineage to Asiatic *Naja,* within which several spitting elapids are nested. The spitting behavior, however, appears to have been lost in this subgenus from the east toward the west (Trans‐Caspia) of Asia.

Our reconstruction of the phylogenetic relationships among Asiatic cobras at interspecific levels recovered a generally well‐supported phylogenetic structure for the Asiatic *Naja*. Our results continue to strongly confirm the monophyly of the Asiatic *Naja* group, in congruence with previous studies based on molecular and morphological evidence (Slowinski & Keogh, 2000; Szyndlar & Rage, 1990; Wüster, 1990; Wüster et al., 2007). Moreover, our phylogenetic reconstructions confirmed that *N. naja* formed a basal lineage relative to other Asiatic *Naja* species, a finding similar to previous studies (Ashraf et al., 2019; Wallach et al., 2009; Wüster et al., 2007). Excluding *N. naja,* which forms the basal clade for our phylogenetic tree, we could distinguish two broad groups of taxa: (a) southeastern Asian cobras including *N. siamensis, N. sumatrana, N. philippinensis, N. samarensis, N. sputatrix,* and *N. mandalayensis,* and (b) western, central, southern, and eastern Asian cobras including *N*. *oxiana, N*. *kaouthia*, *N. sagittifera,* and *N*. *atra*. It appears that this classification is in concordance with patterns of spitting behavior as southeastern Asiatic cobras are known to spit venom, whereas central, western, southern, and eastern Asiatic cobras are regarded as nonspitting or occasional spitters (Wüster et al., 2007).

Our finding of *N*. *kaouthia* and *N. sagittifera* being recovered as sister taxa to each other and in turn sister clades to *N. oxiana* is new. However, Santra et al. (2019) revealed a different phylogenetic position for *N*. *oxiana* using a single mitochondrial marker (16S), incongruent with the results of Wüster et al. (2007), Wallach et al. (2009), Shoorabi et al. (2017), Kazandjian et al. (2020), and the present study. The three *N*. *oxiana* samples used in their study belonged to the eastern population of this species from Himachal Pradesh, India. This incongruence may have been the result of the low variability of this gene and the short length of sequences (488 bp) generated in their study, which failed to fully resolve the phylogenetic relationships of Asian cobras.

### 4.2 Phylogenetic and phylogeographic patterns of *N. oxiana*

In this study, we attempted to present a first view of the phylogeny of the Caspian cobra relative to other Asiatic cobras. At the intraspecies level, our phylogenetic trees revealed no diversification between *N. oxiana's* eastern and western populations, although this finding remains tentative due to the small number of samples from the eastern population. Similarly, no divergence was detected within the western populations in Iran, Afghanistan, and Turkmenistan. The haplotype network showed a large number of common haplotypes between *N. oxiana* populations, suggestive of high rates of gene flow. Moreover, the results of the genetic structure of *N. oxiana* populations are consistent with the phylogenetic trees, showing an integrative genetic group across the entire distribution of the Caspian cobra. Population genetic analyses revealed low haplotype and nucleotide diversities, in line with an earlier study using the D‐loop gene which found low (2–5) mutational steps among haplotypes from northeast of Iran (Shoorabi et al., 2017). However, other intraspecies studies demonstrated several different lineages in some species of Asiatic cobras. For instance, Ratnarathorn et al. (2019) showed that *N. kaouthia* populations diverged into four lineages, suggesting cryptic speciation for *N. kaouthia* populations in northeast of Thailand, affected by climatic or geographical differences. Similarly, two distinct lineages were recovered for *N. atra* in China (Lin et al., 2014).

According to the EBSP results, our demographic history reconstruction exhibited that *N. oxiana's* western ranges expanded between 1,000 and 2,000 years ago during the last age (Meghalayan) of Holocene series (Walker et al., 2019). Furthermore, mismatch distribution provided strong evidence for *N. oxiana*'s unimodal distribution, which is associated with a panmictic population undergone sudden demographic expansion in its evolutionary history (Rogers & Harpending, 1992; Slatkin & Hudson, 1991). In addition, the star‐shaped structure of the haplotype network evidently indicates a sudden expansion (Kerdelhué et al., 2009; Slatkin & Hudson, 1991). Both Tajima's *D* and Fu's *F*s produced significantly negative values for Caspian cobra populations, implying that western populations have at least experienced one expansion phase. A similar pattern was detected for *N. atra* where populations in a vast range of the species' range were affected by no reproductive isolations during the Quaternary glacial period and therefore experienced a sudden range expansion (Lin et al., 2012).

Our molecular dating showed that the divergence between *N. oxiana* and its sister taxa, *N. kaouthia* and *N. sagittifera,* took place during late Pliocene (3.21 Mya in Piacenzian Age), though with wide 95% confidence intervals ranging from early Pliocene (4.48 Mya in Zanclean Age) to early Pleistocene (1.94 Mya in Gelasian Age). At the end of the cold and short Zanclean Age, a warm and wet period of the Piacenzian Age appeared in the northern hemisphere. During the Piacenzian, the ice sheets of Antarctica were less developed and sea levels were about 20 m higher than today. Also, temperature levels were 2–3°C higher. Therefore, we may cautiously infer that at the end of the Zanclean (approximately 3.6 Mya) and with the onset of the warm and wet Piacenzian period (3.60–2.58 Mya), gene flow occurred toward the eastern borders of Afghanistan and Pakistan. The massive Hindu Kush Mountains formed an impassable barrier to *Naja* dispersal. Thus, gene flow was only allowed via the southern parts of Afghanistan, from above the Registan Desert toward Iran's borders and from below this desert toward Balochistan, Pakistan. The occurrence of *N. oxiana* in provinces of Sistan & Baluchistan, Southern Khorasan, and Central Khorasan in Iran supports the proposed hypothesis. Unfortunately, for years, Afghanistan has failed to maintain security for researchers to conduct surveys, resulting in a general lack of scientific data in the region. Hence, we urge caution in concluding that eastern and western *N. oxiana* populations are markedly distinct, as new *N. oxiana* records have lately emerged from southern, western, and northern Afghanistan. Rapid expansion of *Naja* from the east toward Afghanistan and Pakistan and eventually to the east and northeast of Iran is not beyond the bounds of possibility for *Naja* species with such long‐range dispersal potential.

Cyclical climatic fluctuations of the Pleistocene are assumed to have had an integral role in determining the spatial distribution, patterns of genetic diversity and historical demography of a multitude of Palearctic species (Avise, 2000; Hewitt, 2001). Studies on snakes living at high altitudes highlight that mountain vipers have been modified during the glacial climates of Pleistocene and have diversified as a result of the progression and recession of glaciers (Behrooz et al., 2018; Ding et al., 2011). Our findings corroborated that the western population of *N. oxiana* has a genetically uniform structure belonging to a single genetically homogeneous population, while also acknowledging the small number of samples from Afghanistan and Turkmenistan in our study. We posit that *N. oxiana* was not influenced by cold Pleistocene periods and therefore not restricted to glacial refugia, in that it typically inhabits arid and semiarid regions, or rocky, shrub or scrub‐covered foothills and tends to avoid high‐altitude mountain habitats which were subject to past glacial episodes in Iran (Moghimi, 2008, 2010).

Also, no geographical barriers, such as impenetrable mountains, large aquatic structures, or urban areas, have existed to limit the gene flow of *N. oxiana* in this region. We suppose that rapid dispersal of *N. oxiana* from the westernmost extreme of its range toward eastern and northeastern Iran was facilitated by the lack of major dispersal barriers. This ultimately prevented the formation of spatially structured populations in the region. Benefiting from the absence of competitive taxa (e.g., levant viper *Macrovipera lebetina*), the Caspian cobra successfully colonized the entire range from plains and low‐slope foothills of eastern and northeastern Iran to warm and humid plains along the eastern coast of the Caspian Sea.

Finally, although the present study offers the first insight into the phylogeny of *N. oxiana* relative to Asian cobras, we acknowledge that drawing inferences about the phylogeny and phylogeography and delimitation of conservation units based solely on mtDNA, single nDNA genes, or even combined mtDNA and nDNA data may be misleading (e.g., Ballard & Rand, 2005; Ballard & Whitlock, 2004; Rubinoff & Holland, 2005; Shaw, 2002; Wiens et al., 2010). Thus, population genomic studies using large numbers of unlinked nuclear loci are recommended for this species (both western and eastern populations) as well as other Asian cobras.

### 4.3 Conservation units and management propositions

For long, conservation managers have debated how to delineate the minimal units appropriate for conservation management purposes (Amato, 1991; Fraser & Bernatchez, 2001; O'Brien & Mayr, 1991). To fulfill this aim, the term evolutionarily significant unit (ESU) emerged to define special groups of taxa below the species level that warrant specific conservation due to their evolutionary originality (Moritz, 1994; Ryder, 1986). Yet, there exists no consensus on the definition of ESU, and here, we favored the one proposed by Fraser and Bernatchez (2001) as it is focused on isolated populations. They advocated that, for adaptive evolutionary conservation, ESUs are best represented by phylogenetic lineages that exchange gene flow within a species. Therefore, each isolated population of *N. oxiana* qualifies as a fitting ESU for conservation. In addition to being classified as Data Deficient (DD) by the IUCN, the conservation status of *N. oxiana* within the complex group of *Naja* has not yet been assessed and its national conservation has been largely neglected.

Here, we suggest that all *N. oxiana* populations in northeastern Iran, Afghanistan, and Turkmenistan could be treated as a single ESU. Due to the several potential and existing threats facing the Caspian cobra, such as habitat loss caused by agricultural development, horticulture, apiculture, limited movement between population patches, overgrazing and destruction of rangelands, we recommend conservation actions be targeted at this population in Iran. In addition, human‐caused mortality (direct killings), road‐kills due to vehicle collisions, and large‐scale overharvesting by vaccine and serum institutes have also decimated native *N. oxiana* populations in Iran. Harvest of Caspian cobras generally occurs immediately after hibernation, dramatically lowering their chance of mating and successful reproduction. During the last five years, wild Caspian cobras have been captured on a minimum of 400–500 individuals per year to be milked by antivenom manufacturing laboratories under permits issued by the Department of Environment of Iran. Over the past decades, declines in *N. oxiana* populations have been documented throughout its range and local collectors have reported difficulties collecting sufficient numbers from wild populations. This situation is further aggravated by the lack of fundamental knowledge on the ecology and conservation status of venomous snakes in Iran, which poses a major obstacle to their conservation and management (Behrooz et al., 2018). Development of a national action plan is required to ensure long‐term conservation of the Caspian cobra in Iran. This in turn calls for more comprehensive studies to identify the existing and potential threats facing Caspian cobra populations and to clarify the population size, gene flow, population isolation, and future distribution of the species under the impacts of climate change and habitat alteration.

## CONFLICT OF INTEREST

The authors declare to have no competing interests.

## AUTHOR CONTRIBUTIONS

**Elmira Kazemi:** Conceptualization (equal); data curation (equal); formal analysis (equal); writing‐original draft (equal). **Masoud Nazarizadeh:** Data curation (equal); formal analysis (equal); software (equal). **Faezeh Fatemizadeh:** Data curation (equal); formal analysis (equal); methodology (equal); writing‐original draft (equal). **Ali Khani:** Methodology (equal); writing‐original draft (equal). **Mohammad Kaboli:** Conceptualization (equal); formal analysis (equal); methodology (equal); supervision (equal); validation (equal); writing‐review & editing (equal).

## Data Availability

Dataset has been deposited in DRYAD (https://doi.org/10.5061/dryad.r7sqv9s97) and submitted to NCBI (MW172773‐MW172810 for cyt *b* and MW145451‐MW145488 for ND4).

## Appendix A

**Table:**

Species	cyt *b*	ND4	Locality	References
*Vipera ursinii*	FR727046	FR726971	Italy	Ferchaud et al. (2012)
*Vipera renardi ebneri*	FR727095	FR727028	Iran	Ferchaud et al. (2012)
*Montivipera latifii*	MG021073	MG041967	Iran	Behrooz et al. (2018)
*Montivipera wagneri*	AJ275725		Turkey	Lenk et al. (2001)
*Montivipera xanthina*	KJ415303		Turkey	Zinenko et al. (2015)
*Montivipera xanthina*	AJ275724		Turkey	Lenk et al. (2001)
*Montivipera albizona*	AJ275727		Turkey	Lenk et al. (2001)
*Montivipera bornmuelleri*	AJ275726		Lebanon	Lenk et al. (2001)
*Macrovipera lebetina*	KJ415301		Uzbekistan	Zinenko et al. (2015)
*Macrovipera lebetina*	KJ415300		Uzbekistan	Zinenko et al. (2015)
*Macrovipera lebetina*	AJ275713		Turkmenistan	Lenk et al. (2001)
*Bungarus fasciatus*	NC_011393	NC_011393		
*Bungarus flaviceps*	AJ749351.1			Kuch (2007)
*Bungarus bungaroides*	AY973270		Vietnam	Kuch (2007)
*Macrovipera schweizeri*	AJ275715		Greece	Lenk et al. (2001)
*Sistrurus catenatus*	AY223610	AY223648	Texas	Wüster et al. (2008)
*Sistrurus miliarius*	AY223611	U41889	Florida	Wüster et al. (2008)
*Crotalus adamanteus*	AY223605	U41880	Florida	Wüster et al. (2008)
*Crotalus ravus*	AY223609	AY223647	Mexico	Wüster et al. (2008)
*Crotalus simus*	EU624302	AY704885	Costa Rica	Wüster et al. (2008)
*Crotalus tigris*	AY223606	AF156574	Arizona	Wüster et al. (2008)
*Porthidium arcosae*	AF292575	AF292613	Ecuador	Wüster et al. (2008)
*Porthidium lansbergii rozei*	AF393623	AF393623	Venezuela	Wüster et al. (2002)
*Porthidium nasutum*	AY223579	U41887	Costa Rica	Wüster et al. (2008)
*Montivipera raddei*	MG020988	MG042007	Iran	Behrooz et al. (2018)
*Montivipera kuhrangica*	MG021061	MG042036	Iran	Behrooz et al. (2018)
*Montivipera albicornuta*	MG021002	MG041960	Iran	Behrooz et al. (2018)
*Montivipera latifii*	MG021067	MG041959	Iran	Behrooz et al. (2018)
*Porthidium nasutum*	AF292574	AF292612	Ecuador	
*Hemachatus haemachatus*	AF217821		South Africa	Slowinski and Keogh (2000)
*Naja nivea*	AF217827	AY058983		Wüster et al. (2007)
*Naja haje*	DQ897746	DQ897703	Tanzania	Wüster et al. (2007)
*Naja mandalayensis*	MT346710	MT346906		Kazandjian et al. (2020)
*Naja mandalayensis*	MT346709	MT346905		
*Naja mandalayensis*	AF155211		Myanmar	Slowinski and Wüster (2000)
*Naja atra*	JN160649		China	Lin et al. (2012)
*Naja atra*	JN160648		China	Lin et al. (2012)
*Naja atra*	JN160647		China	Lin et al. (2012)
*Naja atra*	JN160646		China	Lin et al. (2012)
*Naja atra*	JN160645		China	Lin et al. (2012)
*Naja atra*	JN160644		China	Lin et al. (2012)
*Naja atra*	JN160643		China	Lin et al. (2012)
*Naja naja*	GQ359506	AY713378	Nepal	Wüster et al. (2008)
*Naja naja*	FR871895		Nepal	
*Naja naja*	EU624299	AY713378	Nepal	Wüster et al. (2008)
*Naja sputatrix*	DQ897734	DQ897691	Indonesia	Wüster et al. (2007)
*Naja sputatrix*	MT346729	MT346921		Kazandjian et al. (2020)
*Naja sputatrix*	MT346730	MT346922		Kazandjian et al. (2020)
*Naja sumatrana*	MT346735	MT346931		Kazandjian et al. (2020)
*Naja sumatrana*	MT346736	MT346932.		Kazandjian et al. (2020)
*Naja sumatrana*	MT346737	MT346933		Kazandjian et al. (2020)
*Naja sumatrana*	AB920240			Supikamolseni et al. (2015)
*Naja philippinensis*	MT346719	MT346915		Kazandjian et al. (2020)
*Naja philippinensis*	MT346718	MT346914		Kazandjian et al. (2020)
*Naja philippinensis*	MT346717	MT346913		Kazandjian et al. (2020)
*Naja philippinensis*	MT346716	MT346912		Kazandjian et al. (2020)
*Naja philippinensis*	MT346715	MT346911		Kazandjian et al. (2020)
*Naja sagittifera*	MT346721	MT346917		Kazandjian et al. (2020)
*Naja sagittifera*	MT346720	MT346916		Kazandjian et al. (2020)
*Naja samarensis*	MT346722	MT346918		Kazandjian et al. (2020)
*Naja samarensis*	MT346723	MT346919		Kazandjian et al. (2020)
*Naja samarensis*	MT346724	MT346920		Kazandjian et al. (2020)
*Naja siamensis*	MT346727	MT346925		Kazandjian et al. (2020)
*Naja siamensis*	MT346728	MT346926		Kazandjian et al. (2020)
*Naja siamensis*	MT346726	MT346924		Kazandjian et al. (2020)
*Naja siamensis*	MT346725	MT346923		Kazandjian et al. (2020)
*Naja siamensis*	AF155214			Slowinski and Wüster (2000)
*Naja siamensis*	AB920242			Supikamolseni et al. (2015)
*Naja oxiana*	MW172773	MW145451	Khorasan Razavi Province	Present Study
*Naja oxiana*	MW172774	MW145452	Khorasan Razavi Province	Present Study
*Naja oxiana*	MW172775	MW145453	Khorasan Razavi Province	Present Study
*Naja oxiana*	MW172776	MW145454	Khorasan Razavi Province	Present Study
*Naja oxiana*	MW172777	MW145455	Khorasan Razavi Province	Present Study
*Naja oxiana*	MW172778	MW145456	Khorasan Razavi Province	Present Study
*Naja oxiana*	MW172779	MW145457	Khorasan Razavi Province	Present Study
*Naja oxiana*	MW172780	MW145458	Khorasan Razavi Province	Present Study
*Naja oxiana*	MW172781	MW145459	Khorasan Razavi Province	Present Study
*Naja oxiana*	MW172782	MW145460	Khorasan Razavi Province	Present Study
*Naja oxiana*	MW172783	MW145461	South Khorasan Province	Present Study
*Naja oxiana*	MW172784	MW145462	North Khorasan Province	Present Study
*Naja oxiana*	MW172785	MW145463	North Khorasan Province	Present Study
*Naja oxiana*	MW172786	MW145464	North Khorasan Province	Present Study
*Naja oxiana*	MW172787	MW145465	Golestan Province	Present Study
*Naja oxiana*	MW172788	MW145466	Turkmenistan	Present Study
*Naja oxiana*	MW172789	MW145467	Turkmenistan	Present Study
*Naja oxiana*	MW172790	MW145468	Turkmenistan	Present Study
*Naja oxiana*	MW172791	MW145469	Turkmenistan	Present Study
*Naja oxiana*	MW172792	MW145470	Turkmenistan	Present Study
*Naja oxiana*	MW172793	MW145471	Turkmenistan	Present Study
*Naja oxiana*	MW172794	MW145472	Afghanistan	Present Study
*Naja oxiana*	MW172795	MW145473	Afghanistan	Present Study
*Naja oxiana*	MW172796	MW145474	Afghanistan	Present Study
*Naja oxiana*	MW172797	MW145475	Khorasan Razavi Province	Present Study
*Naja oxiana*	MW172798	MW145476	Khorasan Razavi Province	Present Study
*Naja oxiana*	MW172799	MW145477	Khorasan Razavi Province	Present Study
*Naja oxiana*	MW172800	MW145478	Khorasan Razavi Province	Present Study
*Naja oxiana*	MW172801	MW145479	Khorasan Razavi Province	Present Study
*Naja oxiana*	MW172802	MW145480	North Khorasan Province	Present Study
*Naja oxiana*	MW172803	MW145481	Golestan Province	Present Study
*Naja oxiana*	MW172804	MW145482	North Khorasan Province	Present Study
*Naja oxiana*	MW172805	MW145483	Golestan Province	Present Study
*Naja oxiana*	MW172806	MW145484	Khorasan Razavi Province	Present Study
*Naja oxiana*	MW172807	MW145485	Golestan Province	Present Study
*Naja oxiana*	MW172808	MW145486	Golestan Province	Present Study
*Naja oxiana*	MW172809	MW145487	Khorasan Razavi Province	Present Study
*Naja oxiana*	MW172810	MW145488	Golestan Province	Present Study
*Naja oxiana*	MT346714	MT346910		Kazandjian et al. (2020)
*Naja oxiana*	MT346713	MT346909		Kazandjian et al. (2020)
*Naja kaouthia*	MK721314		Nakhon Ratchasima	Ratnarathorn et al. (2019)
*Naja kaouthia*	MK721310		Nakhon Ratchasima	Ratnarathorn et al. (2019)
*Naja kaouthia*	MK721295		Thailand	Ratnarathorn et al. (2019)
*Naja kaouthia*	MK721303		Pha Ngan Island	Ratnarathorn et al. (2019)
*Naja kaouthia*	MK721269		Thailand	Ratnarathorn et al. (2019)
*Naja kaouthia*		JF357930		Lukoschek et al. (2012)
*Naja kaouthia*	GQ359507	EU624209	Thailand	Wüster et al. (2008)
*Naja kaouthia*	FR693733			
*Naja kaouthia*	FR693728			

Behrooz et al. (2018); Douglas and Gower (2010); Ferchaud et al. (2012); Kazandjian et al. (2020); Kuch, 2007; Lenk et al. (2001); Lin et al. (2012); Lukoschek et al. (2012); Ratnarathorn et al. (2019); Slowinski and Keogh (2000); Slowinski and Wüster (2000); Supikamolseni et al. (2015); Wüster et al. (2002), Wüster et al. (2007), Wüster et al. (2008); Zinenko et al. (2015).
