# Erratum

**DOI:** 10.1002/ece3.7455

**Published:** 2021-05-25

In “The phylogeny, phylogeography, and diversification history of the westernmost Asian cobra (Serpentes: Elapidae: *Naja oxiana*) in the Trans‐Caspian region,” which was published in volume 11 issue 5 March 2021, the top panel of Figure 2 was missing. Additionally, in the caption the explanation for the color codes incorrectly described purple as representing Golestan and yellow as representing South Khorasan when in fact they should have been the other way round (yellow represents Golestan and purple represents South Khorasan).

The correct Figure 2 and caption are shown below:

**Figure 2:**
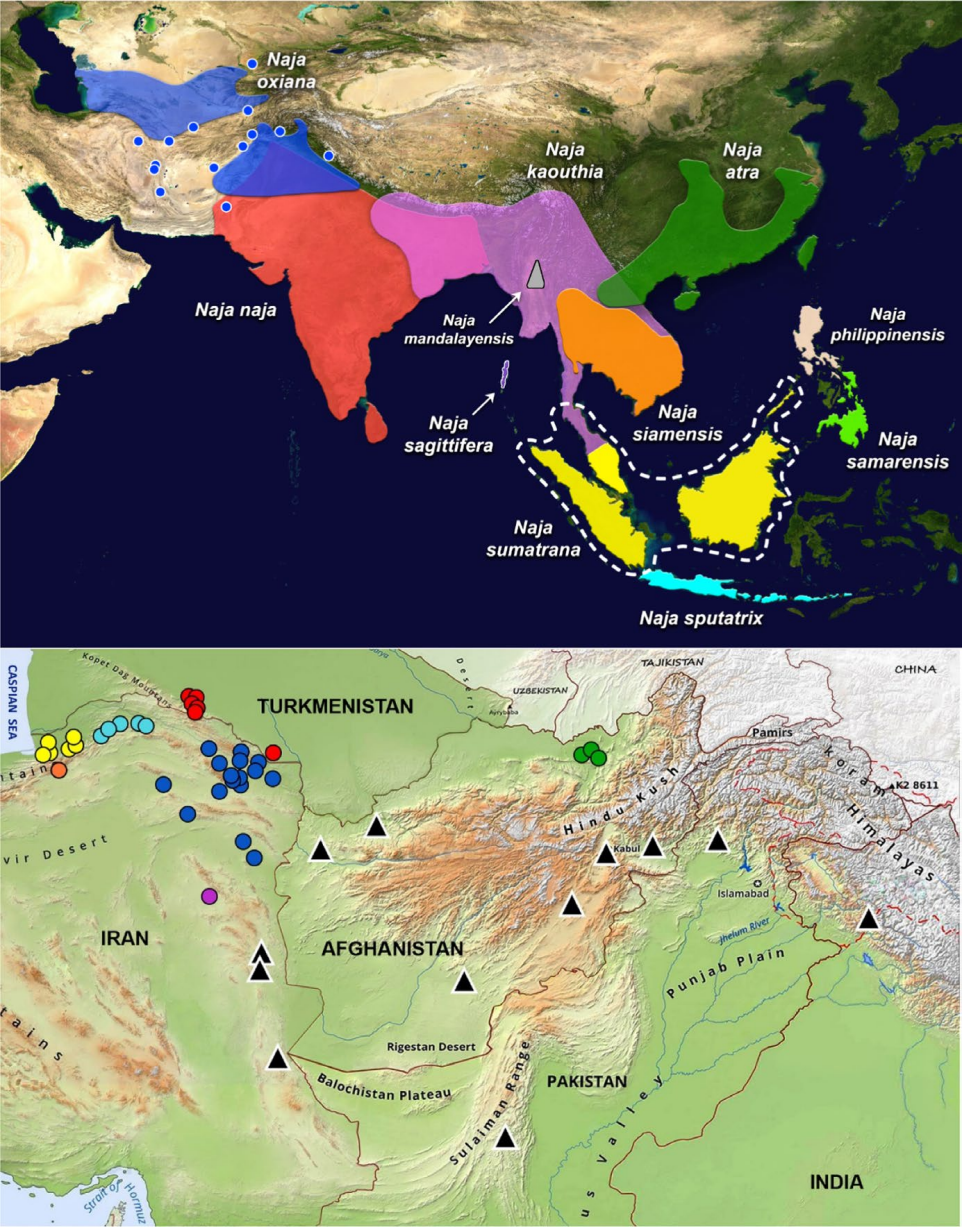
Top: map showing the distribution of the 11 cobra species of Asiatic *Naja*, ranging from Trans‐Caspia to southern and southeastern Asia. Geographic ranges were obtained from the IUCN Red List (IUCN, 2020) and Wüster (1996) and updated for some species based on new occurrence records. Blue circles represent *N. oxiana* occurrence points that lie outside the species' known range. White dotted line represents *N. sumatrana's* range boundary. Bottom: map of sampling localities of the 38 specimens from Iran, Turkmenistan, and Afghanistan. Colors correspond to sampling localities of *N. oxiana*, namely Golestan (yellow), Semnan (orange), Northern Khorasan (light blue), Central Khorasan (dark blue), and Southern Khorasan (purple) provinces of Iran, as well as Turkmenistan (red) and Afghanistan (green). Black triangles once again represent *N. oxiana's* occurrence points that lie outside the species' known range
